# Phylogenetic and Genomic Characterization of Whole Genome Sequences of a Herpes Simplex Virus Type 1 Isolate Identified Genomic Variant Characteristics in a Human Subject with Fulminant Hepatitis

**DOI:** 10.3390/ijms27135640

**Published:** 2026-06-23

**Authors:** Carlo Smirne, Greta Romano, Paolo Ravanini, Maria Grazia Crobu, Antonia Palumbo, Guglielmo Ferrari, Alessio Mercandino, Elena Grossini, Mario Pirisi, Antonio Piralla

**Affiliations:** 1 Department of Translational Medicine, Università del Piemonte Orientale, 28100 Novara, Italy; elena.grossini@med.uniupo.it (E.G.); mario.pirisi@med.uniupo.it (M.P.); 2 Internal Medicine Unit, San Giovanni Bosco Hospital, ASL Città di Torino, 10154 Turin, Italy; 3 Microbiology and Virology Department, Fondazione IRCCS Policlinico San Matteo, 27100 Pavia, Italy; guglielmo.ferrari01@universitadipavia.it (G.F.); antonio.piralla@unipv.it (A.P.); 4 Laboratory of Molecular Virology, Maggiore della Carità Hospital, 28100 Novara, Italy; paolo.ravanini@gmail.com (P.R.); mcrobu@cittadellasalute.to.it (M.G.C.); antonia.palumbo@maggioreosp.novara.it (A.P.); alessio.mercandino@maggioreosp.novara.it (A.M.); 5 Clinical Biochemistry Laboratory, Department of Laboratory Medicine, City of Health and Science University Hospital, 10126 Turin, Italy

**Keywords:** herpes simplex virus 1, acute hepatitis, acute liver failure, viral hepatitis, liver injury, liver function, phylogenesis, molecular sequence annotation, whole genome sequencing, immunosuppression

## Abstract

Herpes simplex virus 1 (HSV-1) is a rare cause of acute hepatitis, especially in patients with chronic immunosuppression. We performed whole-genome HSV-1 sequencing with a metagenomics approach on peripheral blood samples from an Italian case of fatal acute liver failure with high circulating HSV-1 (1,129,900,000 copies/mL), followed by phylogenetic analysis. After multiple sequence alignment, a final dataset of 182 whole-genome sequences was selected. The sequenced HSV-1 strain belonged to a phylogenetic clade isolated in Florida in 2002 (OQ724868.1). A characterization of single nucleotide polymorphisms and indels was performed to determine their effects on the viral genome: only one variant, classified as an indel, was detected with a high impact effect (c.905_906insGTTTT) in the *UL49A* gene, which is known to encode a membrane protein regulating virion morphogenesis, replication and assembly. In addition, this study also detected variants in other genes involved in crucial steps of the HSV-1 life cycle, like alpha-regulation (*US7*), capsid transport (*UL36*) and viral polymerase function (*UL30*). In conclusion, the results of this variant analysis confirmed that in HSV-1 hepatitis, some viral regions may be hotspots for adaptive mutations with a substantial impact on viral replication or immune evasion.

## 1. Introduction

Herpes simplex virus 1 (HSV-1), belonging to the *Herpesviridae* family, is a rare cause of acute hepatitis, first described in 1969. Indeed, this condition has been described to cause acute liver failure (ALF) and death, although it accounts for less than 1% of all ALF cases and less than 2% of all viral-related etiologies [1,2]. This occurrence is reported especially in subjects with chronic immunosuppression, such as recipients of solid organ transplants or subjects affected by systemic lupus erythematosus or rectal cancer, and in pregnant women in their third trimester [1,3,4,5,6]. However, it is noteworthy that around 25% of patients who present with HSV-1 hepatitis are immunocompetent, with several proposed viral and host mechanisms to explain these disseminated infections [1,7,8,9]. Notoriously, the presenting signs and symptoms can be non-specific, thus causing frequent cases of missed or underestimated diagnosis, especially in individuals without risk factors for disseminated infection. This possibly leads to rapid progression to ALF in the absence of early antiviral therapy (whereas, on the contrary, early treatment with acyclovir can decrease reported death or liver transplant rates from 88% to 51%) [1,10].

In this respect, the phylogenetic and genomic characterization of HSV-1, which is already considered crucial for mapping viral evolution, monitoring human migration or viral recombination patterns, and understanding strain-specific virulence or drug resistance mechanisms [11,12,13,14,15], could also have a role in the clinical diagnostics and epidemiology of the aforementioned severe liver manifestations.

HSV-1 displays considerable genetic diversity, and whole-genome sequencing has enabled the reconstruction of its global phylogenetic structure. Early analyses based on complete genomic sequences identified a six-clade structure reflecting geographic origin and human migration history: Clade I encompasses European and North American strains, Clade II comprises East Asian strains, while Clades III–VI are represented by East African isolates [16]. Conventional genotyping of HSV-1 has relied on intragenic sequence polymorphisms in *US2*, glycoprotein G (*gG/US4*), and gI (*US7*). However, the numerous recombination events shaping HSV-1 evolution mean that single nucleotide polymorphism (SNP)-based schemes do not always yield results consistent with phylogroup classification, limiting their discriminatory value [17]. At the functional level, SNP analysis has proven informative for identifying clinically relevant mutations: acyclovir resistance is predominantly caused by SNP changes in the thymidine kinase gene (*TK/UL23*), with over 130 confirmed resistance-associated polymorphisms, followed by mutations in the DNA polymerase gene *UL30* [18]. Furthermore, even when HSV-1 strains do not cluster phylogenetically by clinical phenotype, individual SNPs are considered more likely to influence virulence, as supported by comparative genomic studies [19]. However, the vast majority of HSV-1 genomic epidemiology efforts have focused on strains associated with orolabial, ocular, or encephalitic disease. Data on the genetic diversity and virulence gene profiles of HSV-1 isolates from other specific clinical contexts remain limited, with the paucity of sequenced isolates constraining the investigation of strain-specific factors influencing disease outcome [19]. In this regard, to the best of our knowledge, no studies have specifically characterized HSV-1 strains recovered from cases of hepatitis and/or ALF, leaving the potential contribution of viral genomic features to these severe manifestations entirely unexplored.

Based on these premises, the aim of the present research was to try to shed light on this specific issue by conducting an in-depth study of the isolates from an Italian subject who experienced a dramatic course of fulminant HSV-1 hepatitis.

## 2. Results

### 2.1. Clinical Evaluation of the Index Patient

A 49-year-old Caucasian immunosuppressed woman presented to the Emergency Department complaining of fever, malaise vomit and onset of abdominal pain. The patient rapidly developed an ALF that led to multiple organ dysfunction syndrome and death within six days from admission. The complete timeline of this case is presented in Figure S1, while a more exhaustive description is reported in File S1. Summarizing, the most clinically salient aspect was that this subject—despite not demonstrating a significant anti-HSV-1 antibody response—tested positive for plasma HSV-1 DNA at a very high load (Table 1). As soon as the molecular biology results were available (that is, a few hours before her untimely death), a rescue therapy with intravenous acyclovir was started, with no noticeable change in her clinical course at this point.

### 2.2. Whole Genome Sequencing and Phylogenetic Analysis

**Figure 1:**
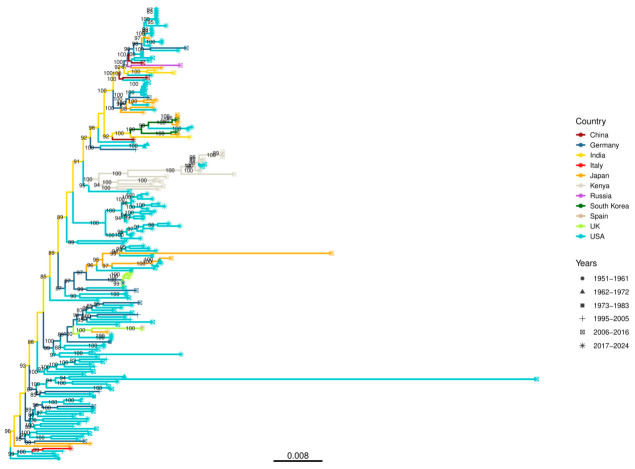
Phylogenetic Maximum Likelihood tree of HSV-1 complete genome sequences. The branches are colored according to origin country; the shape of the nodes is related to sampling date, divided into six 10-year blocks. This graph-based network was generated by aligning the consensus sequences of HSV-1 viral genome with 181 published viral genomes that encompass the known global genetic diversity of HSV-1. HSV-1 strain sequenced here is highlighted in red.

The whole-genome sequencing (WGS) of the strain was obtained with high-throughput sequencing technology. The sequence was multiple-aligned with HSV-1 genomes retrieved from National Center for Biotechnology Information (NCBI) databases. The final dataset contained 182 whole-genome HSV-1 sequences with associated metadata (sampling date and country information) (Figure 1, sky blue). The phylogenetic analysis showed that the Italian HSV-1 strain clustered in a specific branch with the USA clade (strains from Florida of 2002) (OQ724868.1). Overall, the study genome clustered with five closely related HSV-1 genomes originating from the USA (in addition to the aforementioned OQ724868.1, also OP297882.1, OQ724939.1, MN136523.1, and MH999847.1) and circulating between 2005 and 2024. Pairwise genome-wide comparisons with the five closest phylogenetic neighbors showed nucleotide identities ranging from 99.40% to 99.68%, corresponding to 453–835 SNPs across the aligned genome. According to the available metadata, none of these closely related genomes were associated with hepatitis cases but rather originated from ocular or genital clinical specimens.

### 2.3. Sequence Analysis and Variant Calling of the HSV-1 Strain

A characterization of SNPs and insertions/deletions (indels) was conducted to determine their effects on the viral genome. The impact of variant effect was evaluated with a classification in four categories (high, moderate, modifier, low) (Table 2).

A total of 2631 variants were identified; the vast majority were classified as modifiers (87.6%). This category mainly included downstream gene variants (1227; 46.4%) and upstream gene variants (1057; 40.0%), with a smaller contribution from 3′UTR variants (8; 0.3%) and intron variants (12; 0.5%). Variants with moderate impact accounted for 105 (4.0%) of the total and were largely represented by missense variants (103; 3.9%), while conservative in-frame deletions were rare (2; 0.1%). Low-impact variants amounted to 221 (8.4%) and were almost exclusively synonymous variants (220; 8.3%), with a single splice region/intron variant (1; <0.1%). Finally, only one high-impact variant was detected (1; <0.1%), corresponding to a frameshift variant associated with a splice region variant.

**Figure 2:**
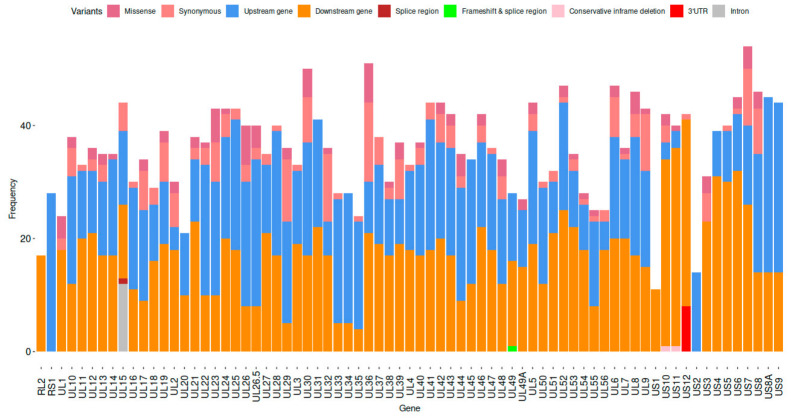
Frequency and functional classification of variants for affected genes of HSV-1.

Figure 2 shows the frequency of these variants in the genes of HSV-1 (*n* = 71) on the basis of the functional classification. Overall, the mean frequency per gene was 10.7 mutations. The highest mutational burden was observed in *US7* (*n* = 54), *UL30* (*n* = 51), and *UL36* (*n* = 50), which displayed the largest number of variants overall. Among the variants with high impact, only *UL49* was affected, reporting one mutation. A total of 42 genes were affected by moderate-impact variants. In detail, *UL36* and *UL26* genes showed the highest number of missense variants (*n* = 7), while *US10* and *US11* were the only ones with a conservative in-frame deletion (*n* = 1). Moderate variants were presented among all the genes and were mainly represented by upstream (range 3–31 mutations) and downstream (range 4–35 mutations) gene variants, with additional contributions from 3′UTR and intron variants. The four exceptions were *RL2*, *US1*, *RS1* and *US2*, which presented downstream and upstream variants only, respectively. Finally, low-impact variants affected 58 out of 73 genes.

## 3. Discussion

Fulminant hepatitis caused by most *Herpesviridae* is a rare but highly fatal form of ALF [4,20,21,22,23]. The most common culprit is HSV, predominantly type 1 or 2 [1,2,24,25]. Specifically, in view of the high mortality and the fact that HSV-1 hepatitis is one of the few causes of fulminant hepatic failure for which potentially effective therapy is available, this pathogen has to be considered early in any case of unexplained ALF. While liver biopsy remains the gold standard for diagnosis (showing characteristic coagulative necrosis and Cowdry type A inclusion bodies), it is often difficult to perform due to severe coagulopathy in patients with ALF. So, if HSV is suspected, HSV DNA detection by PCR on peripheral blood should be requested immediately, as it is a fast and effective screening method, often preferred for its sensitivity over serological testing, especially in immunocompromised subjects. This is because, as reported above, only a rapid start of a specific antiviral therapy, possibly even empirical, can improve the serious prognosis of this illness [26].

Taking these considerations into account, the case described here is not, in itself, entirely exceptional, although it does offer some very interesting insights even from a purely clinical perspective. First of all, to the best of our knowledge, only very few cases with such high levels of circulating viremia have been reported in the literature to date (which, in turn, suggests the presence of an even higher viral load within the liver) (further details about this limited evidence are reported in Table S2) [2,4,5,8,27,28,29]. On the other hand, the complete absence of an IgM antibody response to HSV-1 is noteworthy, especially considering that the patient had been experiencing symptoms for more than a week. The most obvious explanation lies in the immunosuppression caused by the patient’s adjuvant chemotherapy. In detail, carboplatin, a platinum-based agent, usually produces myelosuppression that is characterized, amongst other things, by granulocytopenia, while paclitaxel, the most common taxane, has neutropenia as its main recognized adverse effect [30,31]. However, other contributing factors could also be possible. First of all, this picture could be explained by the known limitations of commercial HSV serology in immunocompromised hosts. Alternatively, it may suggest either a primary infection or a viral reactivation due to an ineffective or delayed host immune response, as for the phenomenon of original antigenic sin, i.e., the cross-reactivity of the HSV-1 and HSV-2 antigens resulting in lower titers of antibodies to the second HSV infection. More prosaically, an analytical error might also be conceivable, in that commonly used serological tests in clinical practice, as in the case of this study, including immunofluorescence, Western blot, and enzyme-linked immunosorbent assays, generally cannot provide highly quantitative and high-throughput results, and many are also unable to make a clear distinction between HSV-1 and HSV-2 [32,33].

However, as mentioned earlier, in our view, the significance of this research extends beyond purely clinical considerations. In fact, using high-throughput sequencing technology, we obtained the whole genome of the strain and evaluated the potential functional impact of genetic variants in this dysfunction syndrome.

The phylogenetic analysis revealed distinct geographic clades within the HSV-1 genome, consistent with previous reports indicating that viral population structure is influenced by geographic isolation and human migration patterns [34,35]. The phylogenetic analysis did not reveal clustering of the study strain within a distinct lineage, as it grouped with HSV-1 strains circulating during the last decade, suggesting that the study strain belongs to a lineage that has been circulating globally during at least the past ten years rather than to a genetically distinct HSV-1 lineage (Figure 1). Moreover, none of these closely related genomes were associated with hepatitis cases, but rather, originated from ocular or genital clinical specimens.

Although a direct association between phylogenetic clustering and increased virulence cannot be established, this finding underscores the relevance of genomic surveillance even for ubiquitous and traditionally conserved viruses such as HSV-1, as distinct viral clades may differ in pathogenicity, immune evasion, or drug resistance [36].

Genome-wide variant analysis revealed a high degree of genetic variability, with more than 2600 variants identified, the vast majority being classified as modifier (*n* = 2304) or low-impact ones (*n* = 221) (Table 2). Specifically, modifier variants are generally located in non-coding regions or affect genes with limited known functional impact, while low-impact variants are assumed to be mostly neutral. Moderate-effect variants that could influence protein function were also found, though to a lesser extent (*n* = 105) (Table 2). These latest variants were detected across 42 viral genes, including key genes involved in viral replication (*UL30*), capsid maturation (*UL26*), alpha-regulation (*US7*) and intracellular transport and egress (*UL36*) (Figure 2). The high mutational burden observed in *UL36* and *UL30*, both central to HSV-1 replication and pathogenicity, raises the possibility that cumulative genomic changes may have contributed to enhanced viral fitness or altered replication kinetics, potentially influencing disease severity.

Only one high-impact variant was identified: an indel in the *UL49A* gene (c.905_906insGTTTT) at position 105483 in the protein-coding region of the open reading frame (ORF), functionally classified as a frameshift and splice region variant (Table 2, Figure 2 green variant). According to analogies with other alphaherpesviruses (e.g., VZV), *UL49A* is involved in syncytia formation, contributing to cell–cell fusion and immune evasion [37], and is a potential component of the virion envelope that may be O-glycosylated [38]. Moreover, this gene was shown to be involved in viral replication in tissue cultures, as HSV-1 mutants with *UL49A* insertions or deletions could not be isolated [34]. However, no direct association between *UL49A* variants and HSV-1 pathogenicity has been reported so far, and the biological significance of this mutation remains to be elucidated.

Among other key genes, *US7*, *UL36*, and *UL30* exhibited the highest numbers of variants (Figure 2): *US7* showed 54 variants, followed by *UL36* (51 variants) and *UL30* (50 variants). From a pathophysiological perspective, *US7* encodes glycoprotein I (gI), a viral membrane component that together with gE forms a complex (gE/gI) that acts as a fragment crystallizable (Fc) receptor and aids cell-to-cell transmission of the virus, thereby avoiding elimination by antibodies [39]. In turn, *UL36* is one of the largest genes in the HSV-1 genome (~3159 amino acids). It encodes VP1/2 (also called pUL36), a large viral tegument protein essential for virion assembly, maturation and release [39]. The function of *UL36* seems to be highly conserved, so significant natural mutations tend to be rarely observed in clinical strains because they would compromise viral replication. However, a study of Desai et al. showed how an induced null mutation in the *UL36* gene can result in accumulation of unenveloped DNA-filled capsids in the cytoplasm of infected cells [39]. Finally, *UL30* encodes the viral polymerase and constitutes the target of antiviral nucleosides (such as acyclovir and foscarnet). Thus, specific variants may confer resistance or altered sensitivity to these specific drugs [40]. Further studies showed that mutations in conserved regions of *UL30*, by altering the replicative capacities of herpesviruses, can reduce the efficacy of other selected antiviral treatments as well [40,41]. Most variants in these genes were located in upstream or downstream regions with predicted modifier effects (Figure 2). In the present study, missense and synonymous variants were detected in all three genes (*US7*: 4 missense, 10 synonymous; *UL36*: 7 missense, 14 synonymous; *UL30*: 5 missense, 8 synonymous). While synonymous variants notoriously do not alter the amino acid sequence, missense variants may change amino acids without necessarily disrupting protein function. Unfortunately, the premature death of the patient precluded the assessment of the in vivo functional consequences of these variants.

The significance of the present report lies in the integration of detailed clinical, virological, and genomic data from a rare manifestation of HSV-1 infection. While HSV-1 hepatitis is a recognized cause of ALF, comprehensive genomic characterization of the infecting strain is rarely available. In the present case, the combination of extremely high viremia, absence of a detectable IgM response, and WGS enabled a more comprehensive investigation of the viral and host factors potentially contributing to disease severity.

Importantly, whole-genome analysis showed that the strain was not associated with a distinct or previously recognized hepatitis-related lineage, but rather belonged to a globally circulating HSV-1 lineage. This finding suggests that severe clinical outcomes such as fulminant hepatitis may occur in the absence of major phylogenetic divergence and are likely the result of a complex interplay between host susceptibility, immune status, and viral genetic background. Furthermore, the identification of numerous variants affecting genes involved in viral replication, intracellular transport, immune evasion, and antiviral response highlights the value of genomic surveillance in improving our understanding of HSV-1 pathogenesis and in generating hypotheses for future functional studies.

Overall, our results indicate that HSV-1 can accumulate numerous variants, primarily in non-coding regions or in genes with limited known function. While most of these are likely neutral or of minor effect, high-impact mutations such as the *UL49A* frameshift may affect viral replication, at least in in vitro models [42]. Variants in genes like *US7*, *UL36*, and *UL30* may also represent regions potentially prone to adaptive mutations, with the hypothetical ability to contribute to immune evasion or antiviral resistance [42,43,44,45].

That being said, it is important to acknowledge that this study also has some important limitations. Specifically, although WGS enabled the reconstruction of the HSV-1 genome and the identification of putatively relevant genetic variants, the sequencing data achieved a genome coverage breadth of approximately 83%. Consequently, a significant fraction of the viral genome could not be reliably characterized, and additional variants may have remained undetected. Moreover, although WGS identified numerous genetic variants, including mutations affecting genes involved in viral replication, intracellular transport, and immune evasion, the pathogenetic significance of these variants remains uncertain. The functional impact of most detected mutations was inferred exclusively through bioinformatic prediction tools, and no in vitro or in vivo experiments were available to validate their biological effects. Therefore, any potential association between the observed genetic profile and the fulminant clinical presentation should be considered hypothesis-generating rather than causative. Finally, although the phylogenetic analysis provided useful information regarding the evolutionary placement of the study strain, it does not support the existence of a specific hepatitis-associated or intrinsically more virulent HSV-1 lineage. Consequently, the phylogenetic findings should be interpreted primarily as evidence of the genetic relationship of the study strain to currently circulating HSV-1 populations rather than as indicators of enhanced pathogenicity.

Nevertheless, despite these shortcomings, we considered it important to perform a genomic characterization of the infecting strain, as studies investigating the whole-genome features of HSV-1 isolates associated with ALF remain, as above reported, extremely scarce. In this respect, the present analysis could provide at least some preliminary insights into the genomic background of a strain associated with this severe clinical presentation and might potentially serve as a starting point for future investigations involving larger case series and more complete genome datasets.

## 4. Materials and Methods

### 4.1. Main Clinical Viral Serology Tests

The assays that were used in the local laboratory for viral serology are amongst those normally used in routine diagnostics during hepatitis workup and included both primary hepatotropic viruses (hepatitis viruses A, B, C, D, and E), and other agents such as herpesviruses and human immunodeficiency virus (HIV). For the purposes of this research, the detailed methods used for HSV detection were as follows: for HSV-1/2 immunoglobulin (Ig) M, LIAISON^®^ HSV-1/2 IgM (DiaSorin, Saluggia, Italy); for HSV-1/2 IgG, LIAISON^®^ HSV-1/2 IgG (DiaSorin); for HSV-1 IgG, LIAISON^®^ HSV-1 IgG (DiaSorin); for HSV-2 IgG, LIAISON^®^ HSV-2 IgG (DiaSorin). Further details on serological tests for HSV are provided in Table S1, panel A.

### 4.2. Main Clinical Viral Molecular Tests

The routine assays used in our laboratory for the molecular detection of HSV-1, HSV-2 and all the other viruses of interest for this research are detailed in Table S1, panel B.

### 4.3. Genomic Analyses

WGS of HSV-1 was performed using a metagenomic approach as previously described [46]. Libraries were prepared with the Illumina DNA Prep kit (Illumina, Inc., San Diego, CA, USA) and sequenced on an Illumina MiSeq System using a MiSeq Reagent Kit v2 (300-cycle format; 2 × 150 bp paired-end sequencing) (Illumina). Sequencing was completed in a 24 h run and generated a total of 7,787,452 raw reads. Sequence data were processed through the Infectious Disease Sequencing Platform (IDseq) pipeline (accessed on 1 October 2024) [47], which was used for quality control filtering, removal of human host reads, reference-guided genome assembly using the HSV-1 reference genome (GenBank accession number: NC_001806.2), and variant calling. Overall, 85.7% of the raw reads passed quality filtering and, after host-read removal, were assembled to reconstruct the complete HSV-1 genome. The sequencing data achieved an average genome coverage depth of approximately 10× and a genome coverage breadth of approximately 83%. Variant calling was performed within the default IDseq metagenomic workflow using samtools mpileup (accessed on 10 October 2024) [47]. The resulting variant call format (VCF) file was directly retrieved from the IDseq output. The following parameters were applied: -aa, reporting all genomic positions including those with zero coverage; -u, generating uncompressed binary call format (BCF) output; -Q 20, excluding bases with a Phred quality score below 20; -d 100000000, setting the maximum per-file read depth to 100,000,000 reads; -L 100000000, setting the maximum number of reads per input file to 100,000,000; and -t AD, reporting allelic depth information for each variant. Variant annotation and prediction of their functional consequences were performed using Single Nucleotide Polymorphism Effect (SnpEff) toolbox version 5.3c (accessed on 1 October 2024) [48].

For phylogenetic analysis, the HSV-1 genome obtained in this study was combined with whole-genome HSV-1 sequences retrieved from the NCBI database. Multiple sequence alignment was performed using Multiple Alignment using Fast Fourier Transform (MAFFT) version 7.525 (accessed on 13 March 2024) [49]. The final dataset comprised 182 HSV-1 whole-genome sequences with associated metadata, including sampling date and country of origin. Maximum-likelihood phylogenetic reconstruction was carried out using IQ-TREE multicore version 2.3.3 (accessed on 12 October 2024) [50]. The robustness of the inferred phylogeny was assessed using the ultrafast bootstrap approximation method with 1000 replicates (accessed on 12 October 2024) [50].

## 5. Conclusions

This case reinforces the importance of early consideration of HSV-1 infection in patients with ALF and highlights the potential value of integrating clinical, virological and genomic data to better understand the wide spectrum of severity typically observed in HSV-1-associated disease. Thus, understanding the functional implications of these genetic variations could potentially inform the development of more effective antiviral therapies that account for the diversity of circulating HSV-1 strains.

## Figures and Tables

**Table 1:** Main patient’s molecular microbiology findings.

Tested Virus	Result
SARS-CoV-2 RNA ^1^	negative
HBV-DNA ^2^	negative
HCV RNA ^2^	negative
HCMV DNA ^2^	negative
EBV DNA ^2^	124 IU/mL
Parvovirus B19 DNA ^2^	985 IU/mL
VZV DNA ^2^	negative
HHV-6 DNA ^2^	279 IU/mL
HHV-8 DNA ^2^	negative
Enterovirus RNA ^2^	negative
HSV-1 DNA ^2,3^	1,129,900,000 cp/mL
HSV-2 DNA ^2^	negative

^1^ nasal swab; ^2^ in plasma; ^3^ a comparable strong positivity was confirmed post-mortem on a second available sample, which had been collected and stored the day before this analysis.

**Table 2:** Classification of variants by their effects.

Impact of Variant Effect	Variant Effect	Number of Variants	Total
High	Frameshift variant and splice region variant	1	1
Moderate	Conservative in-frame deletion	2	105
	Missense variant	103	
Modifier	3′UTR variant	8	2304
	Downstream gene variant	1227	
	Upstream gene variant	1057	
	Intron variant	12	
Low	Synonymous variant	220	221
	Splice region variant and intron variant	1	

## Data Availability

All data generated or analyzed during this study are included in this published article and its Supplementary Information files. The raw sequencing data generated in this study have been deposited in the NCBI Sequence Read Archive (SRA) under BioProject accession number PRJNA1475492.

## Supplementary Materials

The following supporting information can be downloaded at: https://www.mdpi.com/article/10.3390/ijms27135640/s1.

## Abbreviations

The following abbreviations are used in this manuscript:

Ab	antibodies
ALF	acute liver failure
ALP	alkaline phosphatase
ALT	alanine transaminase
aPTT	activated partial thromboplastin time
AST	aspartate transaminase
AU	arbitrary units
AUC	area under the curve
BCF	binary call format
CCM	Center for Disease Prevention and Control
cp	copies
CRP	C-reactive protein
Ct	cycle of threshold
CT	computed tomography
CU	chemiluminescent units
CVC	central venous catheter
DIC	disseminated intravascular coagulation
dRVVT	diluted Russell viper venom time
eGFR	estimated glomerular filtration rate
EA	early antigen
EBNA	Epstein–Barr nuclear antigen
EBV	Epstein–Barr virus
ETI	endotracheal intubation
F	female
Fc	fragment crystallizable
FEU	fibrinogen equivalent unit
FIGO	International Federation of Gynecology and Obstetrics
GE	genome equivalents
GGT	gamma-glutamyl transferase
gE	glycoprotein E
gI	glycoprotein I
HAV	hepatitis A virus
Hb	hemoglobin
HBcAb	anti-hepatitis B core antibodies
HBeAb	anti-hepatitis e antigen antibodies
HBeAg	hepatitis B e antigen
HBsAb	anti-hepatitis B surface antibodies
HBsAg	hepatitis B surface antigen
HBV	hepatitis B virus
HCMV	human cytomegalovirus
HCV	hepatitis C virus
HDV	hepatitis delta virus
HEV	hepatitis E virus
HDL	high-density lipoprotein
HHV	human herpesvirus
HIV	human immunodeficiency virus
HSV	herpes simplex virus
ICU	intensive care unit
IDseq	Infectious Disease Sequencing Platform
Ig	immunoglobulin
IL-1Ra	interleukin-1 receptor antagonist
indel	insertion/deletion
IPF	immature platelet fraction
IU	international units
K	potassium
LA	lupus anticoagulant
LDH	lactate dehydrogenase
LDL	low-density lipoprotein
LLOQ	lower limit of quantification
LOD	limit of detection
M	male
MAFFT	Multiple Alignment using Fast Fourier Transform
MAS	macrophage activation syndrome
MOF	multi-organ failure
Na	sodium
NCBI	National Center for Biotechnology Information
NR	normal range
ORF	open reading frame
PCT	procalcitonin
PLT	platelets
PT-INR	prothrombin time-international normalized ratio
PV	peripheral veins
RFU	relative fluorescence units
SARS-CoV-2	severe acute respiratory syndrome coronavirus 2
SCT	silica clotting time
SNP	single nucleotide polymorphism
SnpEff	Single Nucleotide Polymorphism Effect
SRA	Sequence Read Archive
T0	at time
T120 min	at 2 h
TMA	thrombotic microangiopathy
TS	transferrin saturation
ULOQ	upper limit of quantification
UTR	untranslated region.
VZV	varicella-zoster virus
VCA	viral capsid antigen
VCF	variant call format
WBC	white blood cells
WGS	whole-genome sequencing
