# Supplementary material for: Phylogenetic and Genomic Characterization of Whole Genome Sequences of a Herpes Simplex Virus Type 1 Isolate Identified Genomic Variant Characteristics in a Human Subject with Fulminant Hepatitis
Source: Int J Mol Sci. 2026 Jun 23;27(13):5640. doi: 10.3390/ijms27135640 (PMC13362527; doi:10.3390/ijms27135640)
Supplement: Supplementary file 1 [file ijms-27-05640-s001.zip › Figure S1.pdf]

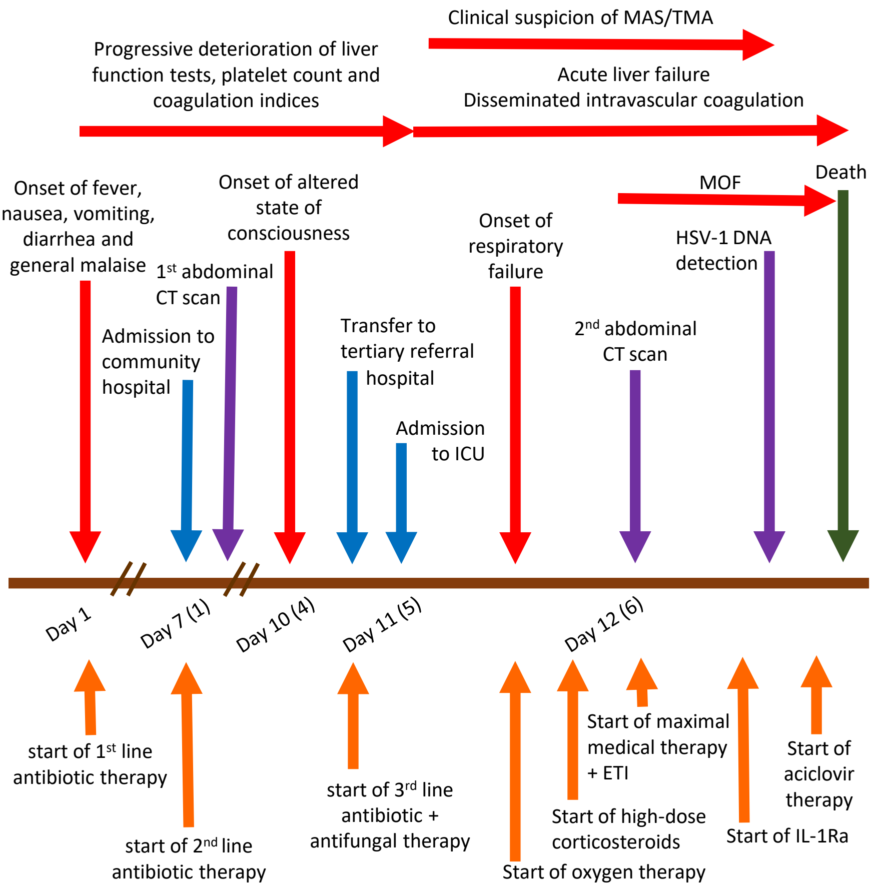

Supplementary Figure S1. Timeline of the clinical case. The number of days is counted both from the onset of symptoms (12 days in total) and the time of the patient's admission (6 days in total, indicated in parentheses, with the last 2 days in the tertiary hospital). Abbreviations: CT: computed tomography; ETI: endotracheal intubation; HSV-1: herpes simplex virus-1; ICU: intensive care unit; IL-1Ra: interleukin-1 receptor antagonist; MAS: macrophage activation syndrome; MOF: multi-organ failure; TMA: thrombotic microangiopathy.
