# Supplementary material for: Phylogenetic and Genomic Characterization of Whole Genome Sequences of a Herpes Simplex Virus Type 1 Isolate Identified Genomic Variant Characteristics in a Human Subject with Fulminant Hepatitis
Source: Int J Mol Sci. 2026 Jun 23;27(13):5640. doi: 10.3390/ijms27135640 (PMC13362527; doi:10.3390/ijms27135640)
Supplement: Supplementary file 1 [file ijms-27-05640-s001.zip › File S1.pdf]

Supplementary File S1. Detailed description of the clinical case.

A 41-year-old patient had a medical history consisting in a pT1b N1 (positive sentinel lymph node) M0 FIGO grade 3 endometrial carcinoma treated with bilateral oophorectomy, with positive peritoneal washing. Based on the stage of the disease, the histology and the patient's good overall condition, adjuvant chemotherapy was started (carboplatin AUC 5 plus paclitaxel 175 mg/m² every 21 days for 6 planned cycles), with adjuvant radiation therapy scheduled upon completion of this treatment. After seven months from surgery, she was cancer-free, in excellent health and had completed five cycles of treatment without any significant side effects.

However, just before starting the scheduled sixth cycle, she started complaining of a remitting fever (up to 40°C) associated with nausea, vomiting and diarrhea, without clear organ-specific symptoms. For this reason, after a few days her general practitioner started her on empirical oral antibiotic therapy with amoxicillin/clavulanate, with no benefit. Due to the worsening of her general condition, about a week after the onset of symptoms she was admitted to the general surgery department of a community hospital following the computed tomography (CT) finding of suspected cholecystitis, accompanied by movement of liver and pancreas cytonecrosis indexes (Supplementary S1).

Supplementary S1. Main patient's laboratory findings. Baseline samples refer to five months prior to the hospitalization described here. The count of days starts from the date of first hospitalization, so the transfer to the tertiary hospital corresponds to day 4. Bold values are outside local laboratory normal ranges.

	**Analyte**	**Baseline**	**1st day**	**3th day**	**4th day**	**5th day**	**6th day**	**Local laboratory NR**
**Blood count**	WBC	7.47	7.61	6.70	**12.56**	**14.70**	7.41	4.50–11.00 × 10⁹/L
	Neutrophils	5.47	7.05	6.12	**11.52**	**13.07**	6.49	1.80–7.70 × 10⁹/L
	Lymphocytes	1.69	**0.46**	**0.45**	**0.73**	1.13	**0.83**	1.00–4.50 × 10⁹/L
	Hb	128	**111**	**105**	**96**	**88**	**73**	117–157 g/L
	PLTs	**411**	220	196	**69**	**39**	**24**	130–400 × 10⁹/L
	IPF	-	-	-	**19.0**	**31.3**	**21.7**	1.0-7.0 %
**Coagulation**	PT-INR	1.03	1.18	**2.49**	**3.45**	**3.57**	**2.84**	0.80–1.20
	PT-INR mixing study T0	-	-	-	-	-	**1.24**	0.80-1.20
	PT-INR mixing study T120 min	-	-	-	-	-	**1.26**	0.80–1.20
	aPTT ratio	0.91	1.10	**2.11**	**2.79**	**2.83**	**3.14**	0.80–1.20
	aPTT mixing study T0	-	-	-	-	-	**1.28**	0.80–1.20
	aPTT mixing study T120 min	-	-	-	-	-	**1.21**	0.80–1.20
	Fibrinogen	270	-	**175**	231	192	**< 35**	180–400 mg/dL
	D-dimer	341	**696**	**14,580**	-	**36,145**	**> 36,145**	0-500 µg/L FEU
	Antithrombin III	-	-	86	-	**65**	**58**	75-120 %
**Renal function**	Creatinine	0.67	0.62	0.94	**1.07**	**1.65**	**2.82**	0.51–0.95 mg/dL
	eGFR	105	106	82	**61**	**36**	**19**	≥ 90 mL/min/1.73 m²
	Na	140	137	**133**	**133**	135	145	134–146 mmol/L
	K	3.5	3.9	4.2	**5.0**	4.3	**6.1**	3.4–4.5 mmol/L
**Liver tests**	AST	17	**418**	**804**	**> 7000**	**> 7000**	**> 7000**	0–40 U/L
	ALT	31	**352**	**617**	**4912**	**4887**	**3623**	0–40 U/L
	GGT	**44**	**153**	**238**	**414**	**404**	**262**	0–40 U/L
	ALP	88	-	-	**212**	**269**	**387**	35–104 U/L
	Bilirubin, total	0.25	0.23	0.33	**2.06**	**2.81**	**2.83**	0.30–1.20 mg/dL
	Bilirubin, direct	0.11	0.19	0.20	**1.80**	**2.49**	**2.59**	0.00–0.25 mg/dL
	Bilirubin, indirect	0.14	**0.04**	0.10	0.26	0.32	0.24	0.10–0.70 mg/dL
	Total protein	80	-	**59**	-	**55**	**57**	64-83 g/L

	Albumin	46	-	-	**29**	**31**	**33**	39.7-49.4 g/L
	Cholesterol, total	**245**	-	-	-	109	-	< 200 mg/dL
	Cholesterol, LDL	**164**	-	-	-	41	-	< 100 mg/dL
	Cholesterol, HDL	68	-	-	-	**< 15**	-	> 65 mg/dL
	Triglycerides	106	-	-	-	147	-	45-170 mg/dL
	Ammonia	-	-	39.2	-	-	-	11-60 µmol/L
**Other tests**	Glucose	86	**153**	-	**112**	**62**	**51**	74–100 mg/dL
	Ferritin	-	-	**63,962**	-	**> 100,000**	**> 100,000**	13–150 µg/L
	Serum iron	66	-	-	-	-	-	50-170 µg/dL
	Transferrin	324	-	**152**	-	-	-	200–360 mg/dL
	TS	15	-	-	-	-	-	20-50% (M); 15-50% (F)
	LDH	**242**	**988**	**2185**	**8164**	**9730**	**> 10,000**	135–214 U/L
	Lactic acid	-	**2.9**	**4.3**	**5.8**	**14.1**	**24.2**	0.5–2.2 mmol/L
	Amylase	-	**204**	**224**	**491**	**787**	**536**	13 – 53 U/L
	Lipase	-	**235**	**252**	**1287**	**1304**	**1336**	0 – 60 U/L
	CRP	0.35	**6.57**	**6.66**	**7.47**	**7.57**	**4.36**	0.00–0.50 mg/dL
	PCT	-	0.40	**2.70**	**3.89**	**3.11**	0.45	< 0.5 µg/L
	IgG	-	-	-	-	855	-	700-1600 mg/dL
	IgM	-	-	-	-	95	-	40-230 mg/dL
	IgA	-	-	-	-	242	-	70–400 mg/dL
**Autoimmunity**	SCT ratio screening for LA	-	-	-	-	1.20	-	0.77-1.20
	dRVVT ratio screening for LA	-	-	-	-	**1.77**	-	0.70–1.20
	Cardiolipin IgM	-	-	-	-	2.3	-	0.0–20.0 CU
	Cardiolipin IgG	-	-	-	-	9.6	-	0.0–20.0 CU
	β2-glycoprotein IgM	-	-	-	-	0.6	-	0.0–20.0 CU
	β2-glycoprotein IgG	-	-	-	-	17.1	-	0.0–20.0 CU

Abbreviations: alkaline phosphatase (ALP); alanine transaminase (ALT); activated partial thromboplastin time (aPTT); aspartate transaminase (AST); C-reactive protein (CRP); chemiluminescent units (CU); diluted Russell viper venom time (dRVVT); estimated glomerular filtration rate (eGFR); female (F); fibrinogen equivalent unit (FEU); gamma-glutamyl transferase (GGT); hemoglobin (Hb); high-density lipoprotein (HDL); immunoglobulin (Ig); immature platelet fraction (IPF); potassium (K); lupus anticoagulant (LA); lactate dehydrogenase (LDH); low-density lipoprotein (LDL); male (M); sodium (Na); procalcitonin (PCT); platelets (PLT); prothrombin time-international normalized ratio (PT-INR); silica clotting time (SCT); at time 0 (T0); at 2 hours (T120 min); transferrin saturation (TS); white blood cells (WBC).

All major viral antibody tests were negative for acute infections (including HAV, HBV, HCV, HIV, EBV, HCMV and VZV); to note also IgM anti-HSV-1/2 were negative at that time. After infectious diseases consultation, she was switched to a second-line antibiotic therapy with piperacillin/tazobactam plus linezolid. However, in the following days the movement of transaminases continued to increase, with associated appearance of predominantly cholestatic jaundice, thrombocytopenia and initial consumption of fibrinogen (Supplementary S1). In light of the worsening clinical picture, including the initial appearance of impaired consciousness, on the fourth day of hospitalization it was decided to transfer the patient to a tertiary hospital.

On new admission, vital parameters were normal except for the presence of fever (39°C) and appropriate tachycardia. The patient was alert, conscious and oriented, but with slight ideomotor slowing without obvious signs of hepatic encephalopathy. Cranial nerves were intact, and muscle strength and reflexes were normal bilaterally, but mild gait and coordination abnormalities were evident. Rigor nucalis was absent. Brain CT scan, abdominal ultrasound and chest X-ray were unremarkable. New blood tests showed a further deterioration

of liver functions tests, platelet count and coagulation indices (Supplementary S1). After multidisciplinary consultation between intensivist, hepatologist, oncologist and infectious disease specialist, the patient was transferred to intensive care unit and a third line antibiotic therapy with meropenem was started, in addition to caspofungin. A new more extended panel of virological tests was performed at this stage, including molecular biology assays for multiple hepatotropic viruses, since the subject was considered to be potentially immunosuppressed given her oncological history. Again, serological assays could not confirm the diagnosis of any acute viral infection (Supplementary S2).

Supplementary S2. Main first-level patient's microbiology findings. Bold values are outside local laboratory normal ranges. (**A**) Serology tests; (**B**) Other tests.

	Test	Result	Local laboratory NR
		(**A**)	
Main hepatitis viruses	HAV IgM	negative	negative
	HAV Ab IgG	**positive**	negative
	HBsAg	< 0.03	0.00-0.05 IU/mL
	HBsAb	< 3	0–11 IU/mL
	HBcAb, IgM	negative	negative
	HBcAb, IgG	negative	negative
	HBeAg	negative	negative
	HBeAb	negative	negative
	HCV Ab	negative	negative
	HDV IgG	negative	negative
	HEV Ab IgM	0.0	< 0.9 index
	HEV Ab IgG	0.1	< 0.9 index
Other hepatitis viruses	HIV Ab	negative	negative
	EBV EBNA IgG	**162**	< 10.0 U/mL
	EBV EA IgG	8.06	< 10.0 U/mL
	EBV VCA IgM	< 10.0	< 10.0 U/mL
	EBV VCA IgG	**93.7**	< 10.0 U/mL
	HCMV Ab IgM	5.4	<18.0 U/mL
	HCMV Ab IgG	**55.0**	<12.0 U/mL
	HSV-1/2 IgM	< 0.5	< 1.0 index
	HSV-1/2 IgG	**1.7**	< 1.0 index
	HSV-1 IgG	**8.3**	< 1.0 index
	HSV-2 IgG	1.4	< 1.5 index
	VZV Ab IgM	0.5	< 1.1 index
Other serological tests	Treponema pallidum Ab IgG	<0.10	0.00-1.00 AU/mL
	Leishmania IgG anti p14	negative	negative
	Leishmania IgG anti p16	negative	negative
		(**B**)	
Other microbiological assays	SARS-CoV-2 rapid antigen test	negative	< 0.01 index
	Serum β-D-glucan	14.34	< 60 pg/mL
	Serum galactomannan antigen test	**0.17**	< 0.16 ratio
	Blood cultures from PV (8 sets)	negative	negative
	Blood cultures from CVC (2 sets)	negative	negative
	T2 bacteria® blood panel	negative	negative
	T2 Candida® blood panel	negative	negative
	Urine culture	negative	negative

Abbreviations: antibodies (Ab); arbitrary units (AU); central venous catheter (CVC); early antigen (EA); Epstein-Barr nuclear antigen (EBNA); Epstein-Barr virus (EBV); hepatitis A virus (HAV); anti-hepatitis B core antibodies (HBcAb); anti-hepatitis e

antigen antibodies (HBeAb); hepatitis B e antigen (HBeAg); anti-hepatitis B surface antibodies (HBsAb); hepatitis B surface antigen (HBsAg); human cytomegalovirus (HCMV); hepatitis C virus (HCV); hepatitis delta virus (HDV); hepatitis E virus (HEV); human immunodeficiency virus (HIV); immunoglobulin (Ig); normal range (NR); peripheral veins (PV); severe acute respiratory syndrome coronavirus 2 (SARS-CoV-2); viral capsid antigen (VCA); varicella-zoster virus (VZV).

The day after a picture of acute liver failure (ALF) became evident in the context of a more generalized multi-organ failure. Moreover, the patient presented a disseminated intravascular coagulation (DIC) pattern with fibrinogen consumption and elevation of D-dimer (> 30,000 μg/L FEU). Concomitantly, a progressive raise of LDH (> 10,000 U/L), ferritin (> 100,000 μg/L) and liver aminotransferases (nearly 200x the upper limits of normal) emerged, together with a worsening bilinear cytopenia (Hb 5.5 g/L, platelets 28 × 10^9/L) (main tests reported in Supplementary S1). A second abdominal CT was performed, showing a large amount of free peritoneal fluid, an enlarged inhomogeneous and hypovascularized liver with right portal vein thrombosis, and a diffuse imbibition of most splanchnic organs (including stomach, gallbladder and small bowel) (Supplementary S3).

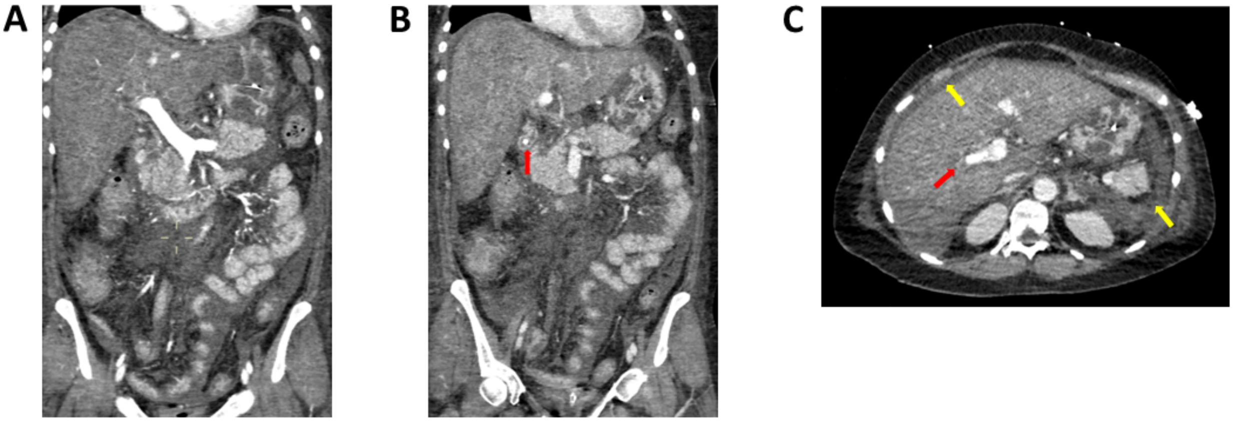

Supplementary S3. Main radiological findings at contrast-enhanced abdominal CT scan. Red arrows show portal thrombosis, while yellow arrows mark ascites or splanchnic imbibition. (**A**) Coronal venous section; (**B**) Oblique coronal section, delayed phase; (**C**) Axial section, delayed phase.

Due to further rapid worsening of the patient's general conditions, the patient was then intubated and started on maximal medical therapy, including inotropic support and multiple blood component transfusions.

To note, the main diagnosis assumed for the entire duration of hospitalization up to this point was septic shock, but other alternative diagnostic hypotheses were also initially formulated. These included, amongst others, thrombotic microangiopathy (TMA) (but no schistocytes were evident at peripheral blood smear and there were no clear signs of hemolysis, so this was reasonably excluded from the hematologist consultant which instead suspected thrombocytopenia and anemia secondary to acute DIC) and a macrophage activation syndrome (MAS) evoked by an infectious or neoplastic trigger (hemophagocityc score was 70-80%, but there was no possibility of a bone marrow biopsy confirmation due to the aforementioned DIC, so high-dose corticosteroids and interleukin-1 receptor antagonist anakinra were ex juvantibus administered, however without any evident clinical benefit).

Finally, in the immediately following hours (that is, less than one day before her untimely death) molecular biology blood tests showed a positive serum HSV-1 DNA at an extremely high viral load (Supplementary S4, please refer also to Table 1 reported in the main text). As soon as this result became available, a

rescue therapy was promptly started with intravenous acyclovir (500 mg, to be repeated once a day due to severe acute kidney failure with eGFR of 19 mL/min), but with no noticeable change in her clinical course at this point.

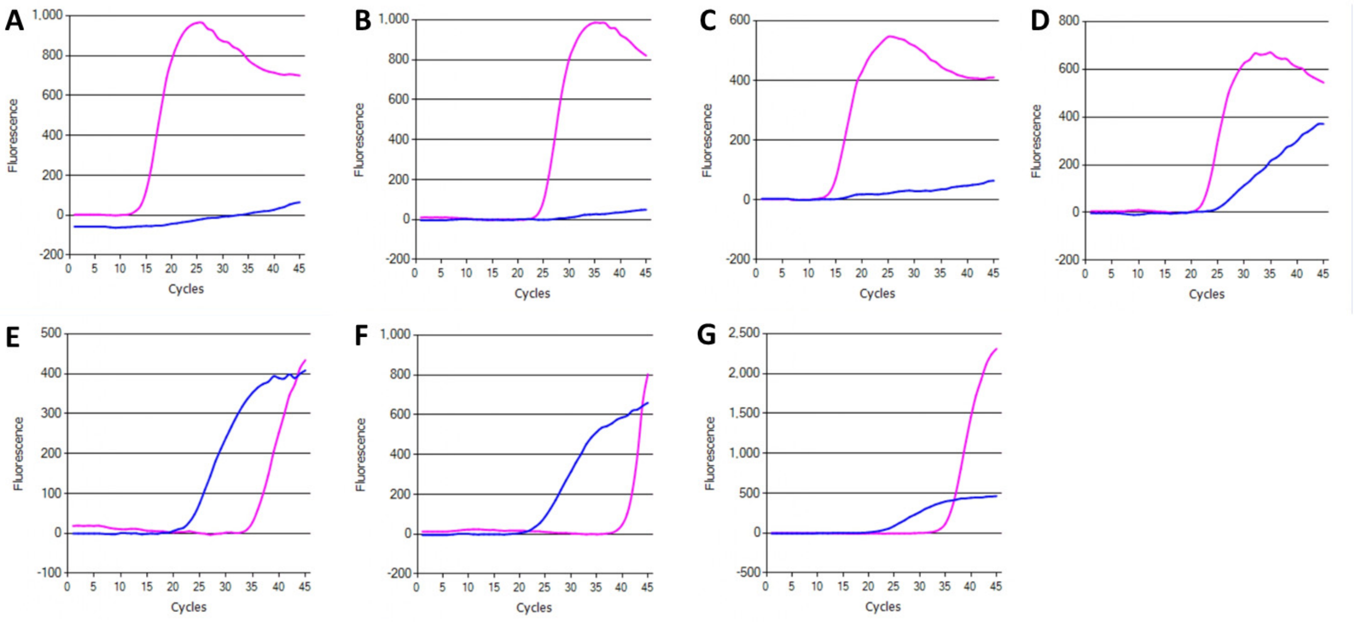

Supplementary S4. Amplification curves of positive plasma quantitative real-time polymerase-chain reaction assays. On the x-axis the cycle numbers are represented. On the y-axis the fluorescence signals are reported on a linear scale, with intensity measurements reported as RFU. Blue lines represent the internal controls. The purple curves represent the viral targets as indicated in the Figure panels. (**A**) Non-diluted HSV-1 amplification. Input template concentration was too high for Ct calculation; (**B**) Diluted (1:1000) HSV-1 amplification. Ct = 24.28; (**C**) Non-diluted HSV-1 amplification (post-mortem confirmation made on a plasma sample still available from the day before the amplification described in point A). Ct as described in point A; (**D**) Diluted (1:1000) HSV-1 amplification with the same methodological specifications as described in point C. Ct = 22.24; (**E**) HHV-6 amplification. Ct = 35.70; (**F**) EBV amplification. Ct = 40.05 (**G**) Parvovirus amplification. Ct = 33.96.
