# Supplementary material for: Phylogenetic and Genomic Characterization of Whole Genome Sequences of a Herpes Simplex Virus Type 1 Isolate Identified Genomic Variant Characteristics in a Human Subject with Fulminant Hepatitis
Source: Int J Mol Sci. 2026 Jun 23;27(13):5640. doi: 10.3390/ijms27135640 (PMC13362527; doi:10.3390/ijms27135640)
Supplement: Supplementary file 1 [file ijms-27-05640-s001.zip › Table S1.pdf]

SupplementaryTable S1. Diagnostic performances of main commercial kits used in this study, as declared by manufacturers. (**A**) Serological tests for HSV-1 and HSV-2; data are presented as medians (interquartile range); (**B**) Molecular diagnostic techniques for all performed viral tests, including HSV-1 and HSV-2 detection.

(**A**)

	Sensitivity [1]	Specificity [1]
**HSV-1/2 IgM**	100.00 (54.07–100.00)	96.46 (92.85–98.57)
**HSV-1/2 IgG**	99.42 (96.82–99.99)	96.84 (91.05–99.34)
**HSV-1 IgG**	94.80 (92.00–96.90)	90.40 (87.80–92.60)
**HSV-2 IgG**	100.00 (94.80–100.00)	99.50 (97.27-99.99)

(**B**)

	Commercial assay kit	LOD	LLOQ	ULOQ
**HSV-1 DNA** [2]	HSV1 ELITe MGB® Kit [4]	250 cp/mL	250 cp/mL	25,000,000 cp/mL
**HSV-2 DNA** [2]	HSV2 ELITe MGB® Kit [4]	119 cp/mL	119 cp/mL	25,000,000 cp/mL
**SARS-CoV-2 RNA** [3]	ALINITY m Resp-4-Plex AMP KIT [5]	30 GE/mL	-	-
**HBV-DNA**	Alinity m HBV [5]	6.72 IU/mL	10 IU/mL	1,000,000,000 IU/mL
**HCV RNA**	Alinity m HCV [5]	8.50 IU/mL	12 IU/mL	200,000,000 IU/mL
**HCMV DNA**	CMV ELITe MGB® Kit [4]	88 IU/mL	88 IU/mL	100,000,000 IU/mL
**EBV DNA**	EBV ELITe MGB® Kit [4]	124 IU/mL	124 IU/mL	1,000,000 IU/mL
**Parvovirus B19 DNA**	Parvovirus B19 ELITe MGB® Kit [4]	125 IU/mL	125 IU/mL	25,000,000 IU/mL
**VZV DNA**	VZV ELITe MGB® Kit [4]	69 cp/mL	69 cp/mL	25,000,000 cp/mL
**HHV-6 DNA**	HHV6 ELITe MGB® Kit [4]	145 IU/mL	145 IU/mL	25,000,000 IU/mL
**HHV-8 DNA**	HHV8 ELITe MGB® Kit [4]	98 cp/mL	98 cp/mL	1.000.000 cp/mL
**Enterovirus RNA** [3]	AllplexTM Respiratory Panel 2 [6]	5400 cp/mL	-	-

Abbreviations: copies (cp); Epstein-Barr virus (EBV); hepatitis B virus (HBV); Epstein-Barr virus (EBV); human cytomegalovirus (HCMV); hepatitis C virus (HCV); human herpesvirus (HHV); herpes simplex virus (HSV); international units (IU); lower limit of quantification (LLOQ); limit of detection (LOD); severe acute respiratory syndrome coronavirus 2 (SARS-CoV-2); upper limit of quantification (ULOQ); varicella-zoster virus (VZV). [1] presented as percentage (95% confidence interval); [2] expressed in cp/mL because these tests have not yet been standardized and there are no IU values available: it is therefore not possible to establish a conversion factor between cp/mL and IU/mL [3] qualitative assay; [4] ELITechGroup, Turin, Italy; [5] Abbott Laboratories, Abbott Park, IL, USA; [6] Seegene Inc., Seoul, South Korea.
