# Supplementary material for: Phylogenetic and Genomic Characterization of Whole Genome Sequences of a Herpes Simplex Virus Type 1 Isolate Identified Genomic Variant Characteristics in a Human Subject with Fulminant Hepatitis
Source: Int J Mol Sci. 2026 Jun 23;27(13):5640. doi: 10.3390/ijms27135640 (PMC13362527; doi:10.3390/ijms27135640)
Supplement: Supplementary file 1 [file ijms-27-05640-s001.zip › Table S2.pdf]

Supplementary Table S2. Levels of circulating HSV-1 viremia in fulminant hepatitis cases reported in the literature (updated June 2026)

Peak HSV-1 DNA, copies/mL	Blood component	Gender	Age	Immunosuppression	Publication year	Reference
2.1×10^6	plasma	M	62	no	2025	[2]
14×10^6	plasma	M	49	yes	2022	[4]
14×10^6	plasma	M	67	yes	2022	[4]
758×10^6	plasma	M	71	no	2021	[5]
94×10^6	plasma	M	65	yes	2021	[5]
1.8×10^9	plasma	M	27	no	2019	[8]
$35 x 10^6$	plasma	F	19	yes	2015	[27]
25.1×10^9	serum	F	26	yes	2011	[28]
1.2×10^9	serum	F	39	no	2008	[29]
